# Direct oligonucleotide sequencing with nanopores

**DOI:** 10.12688/openreseurope.13578.1

**Published:** 2021-05-12

**Authors:** Sachin Chalapati, Conor A Crosbie, Dixita Limbachiya, Nimesh Pinnamaneni

**Affiliations:** 1 Helixworks Technologies, Environmental Research Institute, University College Cork, Cork, T23 XE10, Ireland

**Keywords:** ssDNA, RNA, oligonucleotide, oligo, single-stranded DNA, phosphorylation, T4 PNK, ligation, AMX, T4 DNA ligase, sequencing, ONT, MinION, Guppy, BLASTN, nanopore, helicase, dsDNA, ssDNA, basecalling, squiggle

## Abstract

Third-generation DNA sequencing has enabled sequencing of long, unamplified DNA fragments with minimal steps. Direct sequencing of ssDNA or RNA gives valuable insights like base-level modifications, phosphoramidite synthesis yield estimates and strand quality analysis, without the need to add the complimentary strand. Direct sequencing of single-stranded nucleic acid species is challenging as they are non-compatible to the double-stranded sequencing adapters used by manufacturers. The MinION platform from Oxford Nanopore Technologies performs sequencing by passing single-strands of DNA through a layer of biological nanopore sensors; although sequencing is performed on single-strands, the recommended template by the manufacturer is double-stranded. We have identified that the MinION platform can perform sequencing of short, single-strand oligonucleotides directly without amplification or second-strand synthesis by performing a single annealing step before library preparation. Short 5’ phosphorylated oligos when annealed to an adapter sequence can be directly sequenced in the 5' to 3' direction via nanopores. Adapter sequences were designed to bind to the 5’ end of the oligos and to leave a 3’ adenosine overhang after binding to their target. The 3’ adenosine overhang of the adapter and the terminal phosphate makes the 5’ end of the oligo analogous to an end-prepared dsDNA, rendering it compatible with ligation-based library preparation for sequencing. An oligo-pool containing 42,000, 120 nt orthogonal sequences was phosphorylated and sequenced using this method and ~90% of these sequences were recovered with high accuracy using BLAST. In the nanopore raw data, we have identified that empty signals can be wrongly identified as a valid read by the MinION platform and sometimes multiple signals containing several strands can be fused into a single raw sequence file due to segmentation faults in the software. This direct oligonucleotide sequencing method enables novel applications in DNA data storage systems where short oligonucleotides are the primary information carriers.

## Plain language summary

Traditional DNA sequencing methods need the target DNA to be double-stranded. The MinION platform from Oxford Nanopore Technologies performs sequencing by analyzing the voltage fluctuations while passing a single strand of DNA/RNA through a nanopore. In the current method, single-stranded DNA strands were modified to mimic double-stranded DNA by modifying their ends. The 5' end of the DNA strands are converted into a double strand to facilitate the attachment of the sequencing adapters. To our surprise, sequencing with nanopores did not require the strands to be double-stranded. The modifications of the DNA ends are sufficient to facilitate sequencing of single-stranded nucleic acids. A pool of short, single-stranded DNA molecules was successfully sequenced using this method. The raw data from the nanopore sequencer has shown that the internal software was unable to properly splice the sequence data in some instances. The method to sequence short, single-stranded DNA (oligonucleotides) directly without making them double stranded could be applied in DNA-data storage applications.

## Introduction

DNA sequencing has become a staple tool in biology, has become affordable and more accessible to small labs and individual researchers in the past decade. Oxford Nanopore Technologies (ONT), with its biological nanopore-based sequencing technology, has opened the market wide open by releasing a $1000 sequencing platform – The MinION ^1^. The MinION platform has the capability to sequence both amplified and non-amplified double-stranded DNA (dsDNA) ^2^ and direct RNA in the 3’ to 5’ direction using Poly-A tail capture ^3^. Direct sequencing of short, single-stranded oligonucleotides has been regarded as a challenge due to nanopore chemistry, pore design and basecalling ^4^. However, attempts have been made to overcome these challenges by producing long templates consisting of concatemeric repeats of short target sequences ^5^. Direct sequencing of short, single-strand nucleic acid species without a polymerase or ligation step has not been evident in previous research ^6^. In this article we propose a method to perform direct sequencing of short single strand oligonucleotides that can be leveraged by different applications such as DNA-based data storage systems and direct RNA sequencing.

**Figure 1.**
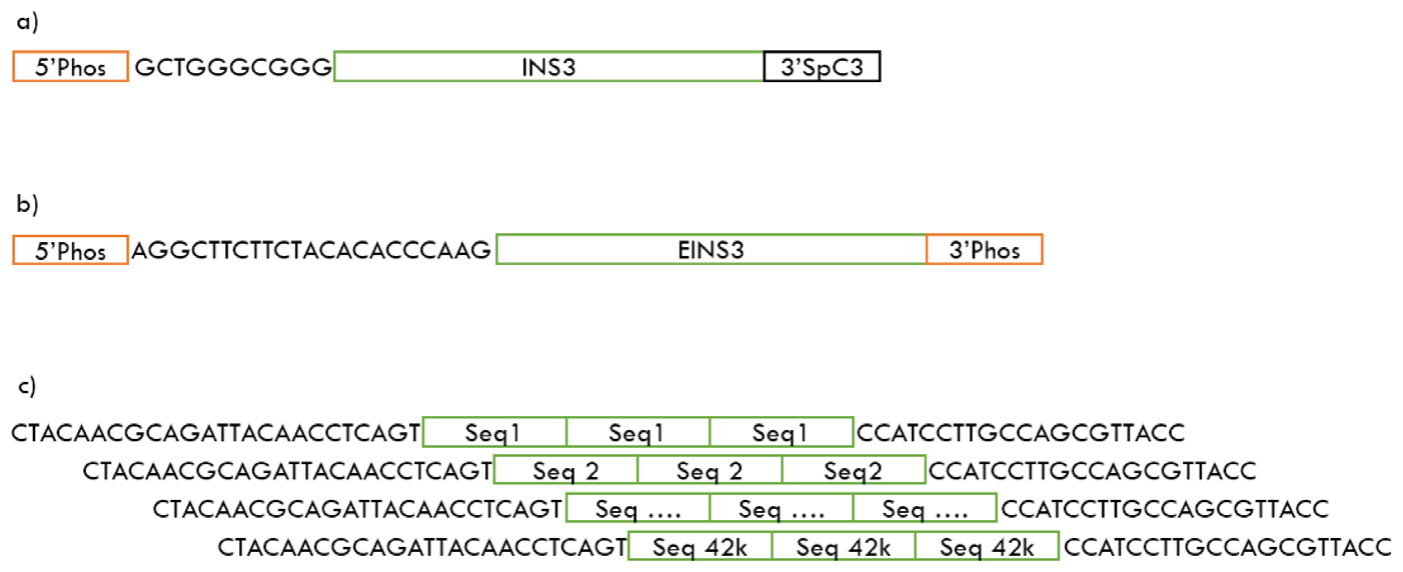
Sequencing templates **a**) INS3 oligo (175 nt) containing a 5’ phosphate and 3’ C3 spacer **b**) EINS3 oligo (200 nt) containing a 5’ phosphate and 3’ terminal phosphate **c**) 3xr6 oligo-pool containing 42,000 unique oligonucleotides of 120 nt length, each of these oligo contain a 3x repetitive region of a 25-nt orthogonal sequence. All sequences in the 3xr6 oligo-pool contain 5’ and 3’ priming regions.

In DNA-based data storage systems, oligonucleotides are information carriers and rapid sequencing of oligos is needed to extract the encoded data ^7^. Several encoding and compression techniques are used to design oligonucleotides for DNA data storage purposes to increase the data capacity and to deal with amplification and sequencing issues. The possibility to sequence oligonucleotides directly without performing a PCR step or performing a ligation step enables users to design the oligos to have only one priming region on the 5' end, freeing up nucleotide space used for the reverse priming region. This increases the encoding space available to the users and opens possibilities for new encoding schema and DNA data storage architectures. In this work, we propose a method that is incredibly fast when compared to PCR-based sequencing strategies ^8^ with hands-on time as low as five minutes and sequencing time of just 20 minutes for pre-phosphorylated oligos like INS3 and EINS3 shown in Figure 1. We have identified that the Oxford Nanopores MinION platform is capable of sequencing single-strand templates directly without the need for a complementary strand or to have a spacer strand to increase the strand length.

**Figure 2.**
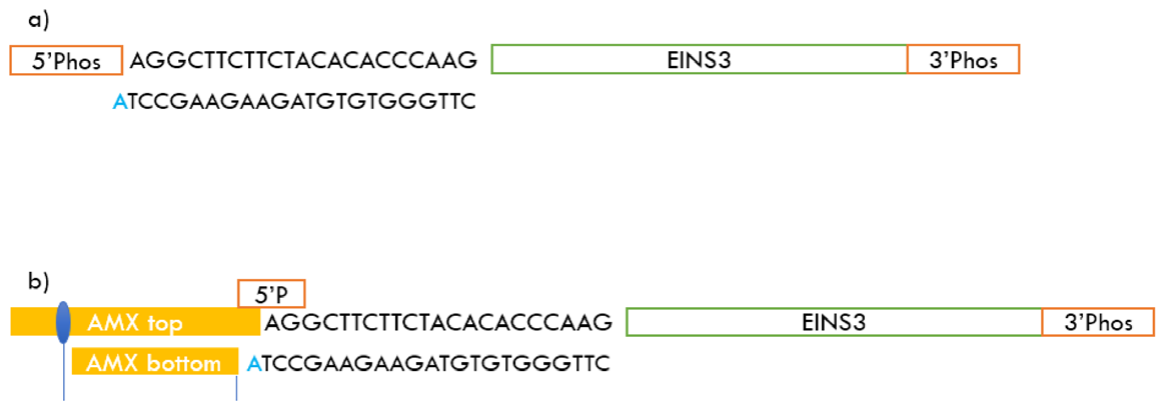
Annealing and ligation steps **a**) Annealing of the adapter to the 5’ end of the EINS3 strand and leaving an adenosine overhang **b**) Ligation of the AMX sequencing adapter to the adapter + EINS3 strand.

In this work, we solve this challenge of direct oligonucleotide sequencing by performing a simple annealing step before the library preparation step. The setup starts with a phosphorylation step using T4 Polynucleotide Kinase (PNK) that adds a 5’ phosphate to the target oligos. The phosphorylated oligos are annealed to an adapter sequence that binds to their 5’ end. The adapter sequences are designed to have a melting temperature of ~65°C to the 5’ end of the target oligos; and when annealed to their targets, the adapter strands have an adenosine overhang at their 3’ end, as detailed in Figure 2. In the annealed state with the adapter sequence, the 5’ end of the oligos are analogous to an end-prepared dsDNA and are compatible with the AMX sequencing adapters form the ligation sequencing kit (LSK-109) offered by ONT ^9^. The sequences used to implement the method and related protocols are discussed in the next section.

## Methods

### Materials and oligonucleotides

Three sets of oligonucleotides were used in this study: INS3, EINS3 and 3xr6. The INS3 and EINS3 sequences were procured from IDT from their Ultramer manufacturing line, both sequences were 5’ phosphorylated at synthesis. INS3 has a 3’ C3 spacer terminator and EINS3 has a 3’ phosphate that were also added during manufacturing. An oligo pool (3xr6) containing 42,000 unique, 120-nt sequences was procured form Twist Biosciences. The annealing adapters INS3 RC, EINS3 RC and ArcFP were procured form IDT and these oligos did not contain any modifications. All the oligos procured from IDT were normalized to 100 uM concentration in TE buffer. The 3xr6 oligo pool was normalized to 10 ng/ul concentration in TE buffer. Please refer to Supplementary Table 1 (see *Extended data*) for sequence data and reagent details ^10^. A step-by-step version of the protocol is available at protocols.io: https://dx.doi.org/10.17504/protocols.io.bt84nryw.

### Normalization and Qubit analysis

The 3xr6 oligo pool was normalized to 0.25 uM in TE buffer by calculating the average molecular weight of the individual oligos and the final concentration was verified using Qubit ssDNA Assay Kit (Cat# Q10212) on a Qubit 4 instrument. The INS3 and EINS3 oligos were diluted to 0.5 uM concentration using TE buffer. The INS3 RC, EINS3 RC and the ArcFP oligos were diluted to their 1 uM working concentrations.

### Phosphorylation and sequencing of 3xr6

The 3xr6 oligo pool was phosphorylated using T4 PNK kinase from NEB. The phosphorylation was carried out at 2 picomole template concentration for 30 minutes at 37°C for 30 minutes followed by a heat inactivation step at 65°C for 20 minutes. The phosphorylated oligo pool was reconcentrated using Monarch spin columns from NEB using the manufacturers oligo cleanup protocol and eluted in TE buffer. The elute from the spin wash was three-way split for a triplicate sequencing run. 1 picomole of the annealing adaptor (ArcFP) was added to each of the triplicate and the temperature was raised to 94°C for 2 minutes for strand denaturation and the mixture was slowly cooled to room temperature for target binding. The sequencing adaptor - AMX from ONT and the Blunt/TA mastermix from NEB were added to each of the triplicates to carry out the ligation reaction between the sequencing adaptor and the oligo pool. The final mixtures were loaded on to three MinION flow cells and the sequencing was performed for 4 hours.

### Library preparation and sequencing of INS3 and EINS3

A triplicate of 0.25 picomoles of INS3 mixed with 0.5 picomoles of INS3 RC in nuclease free water were denatured at 94°C for 2 minutes and slowly cooled to room temperature. The same setup is followed for EINS3 and EINS3 RC with final reaction volumes of 3 ul. The sequencing adaptor (AMX) and Blunt/TA mastermix were added to the reaction tubes to facilitate the ligation reaction. Three flow cells were used for the INS3 triplicate sequencing run and the flowcells are washed as per the manufacturers protocol and the three flow cells were again used for the EINS3 triplicate run. Each of the triplicate sequencing runs were carried out for 20 minutes.

### Sequencing run and basecalling

The sequencing runs were carried out on r9.4 MinION flow cells from ONT at default settings on MinKNOW for the ligation sequencing kit. The manufacturer recommends the DNA to be cleaned before loading on to the flowcell, but in this study the oligos were directly loaded alongside the ligase without a wash step. The sequencing runs were carried out for 4 hours in the case of the 3xr6 oligo pool and for 20 minutes each in the case of INS3 and EINS3. The fast5 raw signal files were basecalled on a laptop with dedicated Nvidia GPU (RTX 2060) using GPU-Guppy (ver. 3.5.2) in the high accuracy mode ^11^. The generated fastq files were binned into pass or fail folders based on their q-scores. Only the reads that have passed the q-score threshold were analyzed.

### BLASTN analysis and FAST5 visualization

BLAST-N program from NCBI was run locally with different modes. The default mode was run for identifying longer matches, whereas the short regions were identified using the ‘blastn-short’ flag. 99-percentile matches were calculated based on their high identity and accuracy scores to the reference sequences. The blast analysis was performed on a 6-core Intel CPU with 12 threads. The fast5 raw data was visualized with HDFView (Ver 3.1.0) to identify and understand the morphology of the single-stranded DNA sequencing.

## Results

### Sequencing of INS3

The flowcells 1, 2 and 3 yielded 23248, 15917 and 21052 reads, respectively, after 20 minutes of sequencing and the reads were basecalled. A total of 289, 483 and 255 reads from flowcells 1 to 3 passed the default q-score filter. The sequencing and BLASTN results with e-value filter are shown in Table 1.

**Table 1.** INS3 sequencing results.

	*Total* *reads*	*Total reads* *passing* *q-score* *filter*	*Filter* *pass %*	*All* *significant* *matches*	*Significant* *matches to* *INS3 (e value* *< 1e-65)*	*Significant matches* *to AMXpINS3* *(ACGTATTGCTGCTGGGCGGG)* *"blastn-short"*
*Flowcell 1*	23248	289	1.24	281	4	693
*Flowcell 2*	15917	483	3.03	414	11	820
*Flowcell 3*	21052	255	1.21	228	4	635

### Sequencing of EINS3

The flowcells 1, 2 and 3 yielded 23174, 30238, and 30051 reads, respectively, after 20 minutes of sequencing and the generated reads were basecalled. A total of 6489, 5041 and 3826 reads from flowcells 1 to 3 passed the default q-score filter. The sequencing and BLASTN results with e-value filter are shown in Table 2.

**Table 2.** EINS3 sequencing results.

	*Total* *reads*	*Total reads* *passing* *q-score* *filter*	*Filter* *pass %*	*All* *significant* *matches*	*Significant* *matches to* *eins3 (e value* *< 1e -75)*	*Significant matches* *to AMXpEINS3* *(ACGTATTGCTAGGCTTCTTC)* *"blastn-short"*
*Flowcell 1*	23174	6489	28.00	5922	143	10363
*Flowcell 2*	30238	5041	16.67	4767	53	10264
*Flowcell 3*	30051	3826	12.73	3699	34	8966

### Sequencing of 3xr6

The flowcells 1, 2 and 3 yielded 60299, 48432 and 42434 reads, respectively, after four hours of sequencing followed up by basecalling. A total of 28869, 13299 and 10268 reads from flowcells 1 to 3 passed the default q-score filter. The sequencing and BLASTN results with e-value filter are shown in Table 3.

**Table 3.** 3xr6 sequencing results.

	*Total* *reads*	*Total reads* *passing* *q-score* *filter*	*Filter* *pass %*	*At least 1* *significant* *match vs* *42k targets* *(e value* *< 1e -15)*	*At least 1* *significant* *match* *vs 42k* *addresses (e* * value < 1e* * -02) "blastn-* *short"*	*Significant matches* *to AMXpFP* *(ACGTATTGCTCTACAACGCA)* *"blastn-short"*	*Potential fusion* *matches FPpRP* *(CAGCGTTACCCTACAACGCA)* *"blastn-short"*
*Flowcell 1*	60299	28869	47.877	25177	31436	88862	78
*Flowcell 2*	48432	13299	27.46	12146	19128	35789	39
*Flowcell 3*	42434	10268	24.2	10374	16678	30605	25
*Combined*	151165	52436	34.69	32028	37265	155256	142

### Data analysis

BLASTN analysis of INS3, EINS3 and 3xr6 are shown in Figure 4, Figure 5 and Figure 6, respectively, which show the total number of reads that pass the quality threshold and the number of significant matches that are found. For INS3 and EINS3, the input query is their full sequence, and the total number of significant matches are plotted. The significant matches are searched with default BLASTN parameters. High-quality matches are also plotted with a 99-percentile label in the figures; these matches show very-high identity to the search query. Refer to the BLASTN output files available as underlying data (see *Data availability* section).

The data from 3xr6 sequencing run is analyzed with BLASTN to search for the full-length sequences along with the short 25-nt orthogonal sequence. The 25-nt orthogonal sequences used during the design of 3xr6 oligo-pool are taken directly from a published source ^12^. Each of the 42,000 (120-nt) oligos in the 3xr6 oligo pool contain a unique 25-nt orthogonal sequence that is repeated three times within the same strand (3x25-nt = 75-nt). All sequences in the 3xr6 oligo pool contain the same forward and reverse priming regions for PCR-compatibility. The 3xr6 oligo-pool was not amplified prior to sequencing in this study.

## Discussion

The technique of modifying the 5’ end of an oligo to make it compatible for nanopore sequencing has resulted in some interesting insights into the sequencing mechanism. The helicase-bound sequencing adapter (AMX) has a thymine (T) overhang on its top-strand and an oligo with a 5’ phosphate and a short (10-nt) 5’-end double-strand region with an adenosine (A) overhang that can facilitate sequencing. The biological nanopore used by ONT’s MinION system can process a single-stranded template without the impeding force provided by the complementary strand. Although the helicase modifications and the nanopore mechanism is proprietary, we believe that the voltage gradient was the driving force behind the strand translocation through the R9.4.1 nanopore flowcells. The helicase may or may not be functional but could be impeding the strand translocation and slowing it enough to perform a high-resolution scan through a k-mer.

We have also identified that the oligonucleotides can even be 3’ unblocked if they do not contain a terminal thymine (T), which can lead to circularization or concatenation products. The INS3 and EINS3 sequences are designed using human insulin gene template ^13^ and manufactured by phosphoramidite process. Both INS3 and EINS3 strands have a 5’ phosphate and 3’ blocker molecules added during their synthesis. The oligos in the 3xr6 oligo-pool are phosphorylated using T4 PNK before annealing to its adapter sequence. The 3’ end of the oligos in 3xr6 are unprotected, unlike INS3 and EINS3.

**Figure 3.**
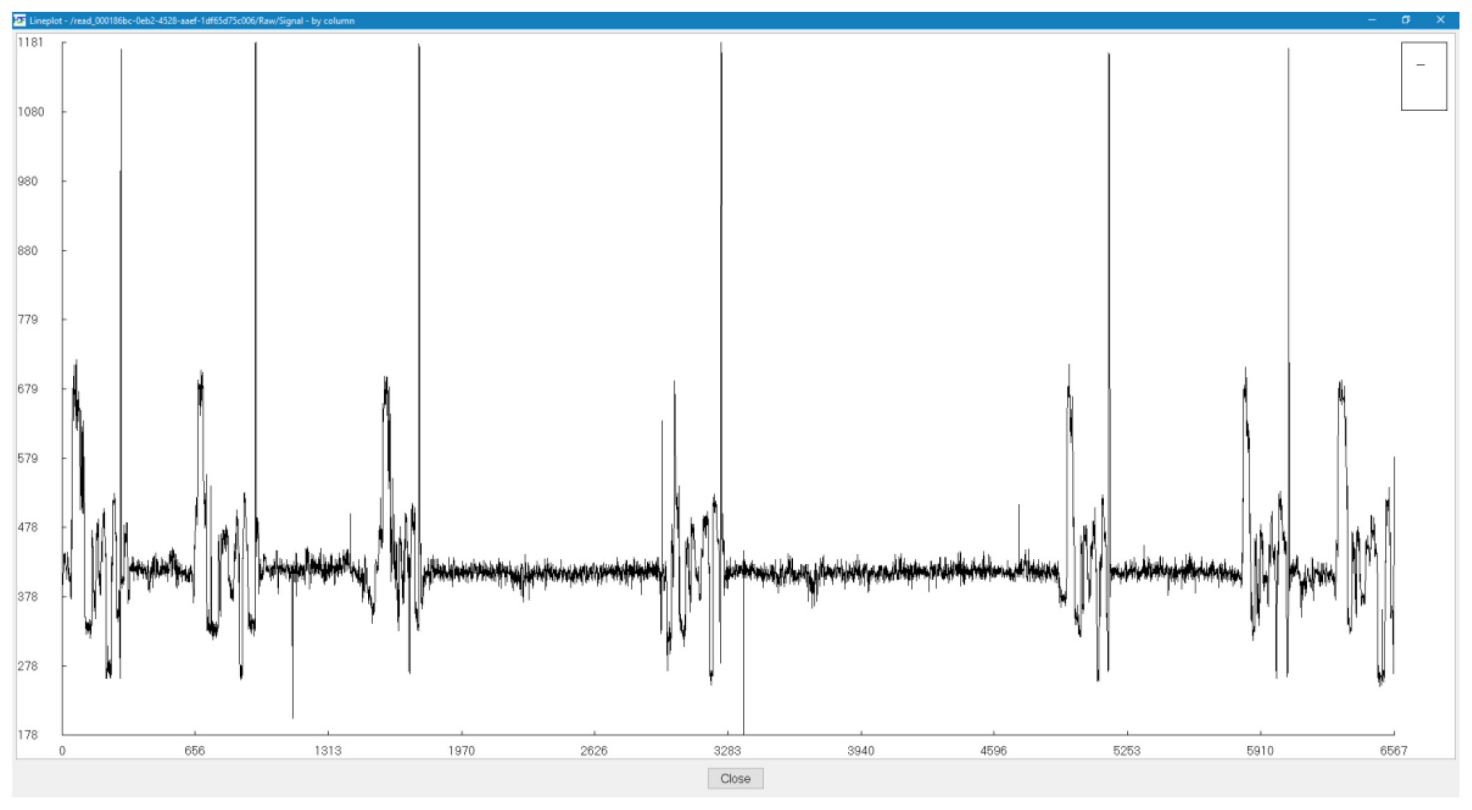
Visualization of a low-scoring INS3 (flowcell 1) read.

We have observed that several of the reads that are generated by the sequencer are empty without any viable signal data as visualized in Figure 3 using HDFView (Ver 3.1.0) ^14^, these reads contain several stall events and helicase dissociation events that are evident from the spike signals. Reads that pass the Guppy quality check are basecalled and analyzed using BLASTN ^15^.

**Figure 4.**
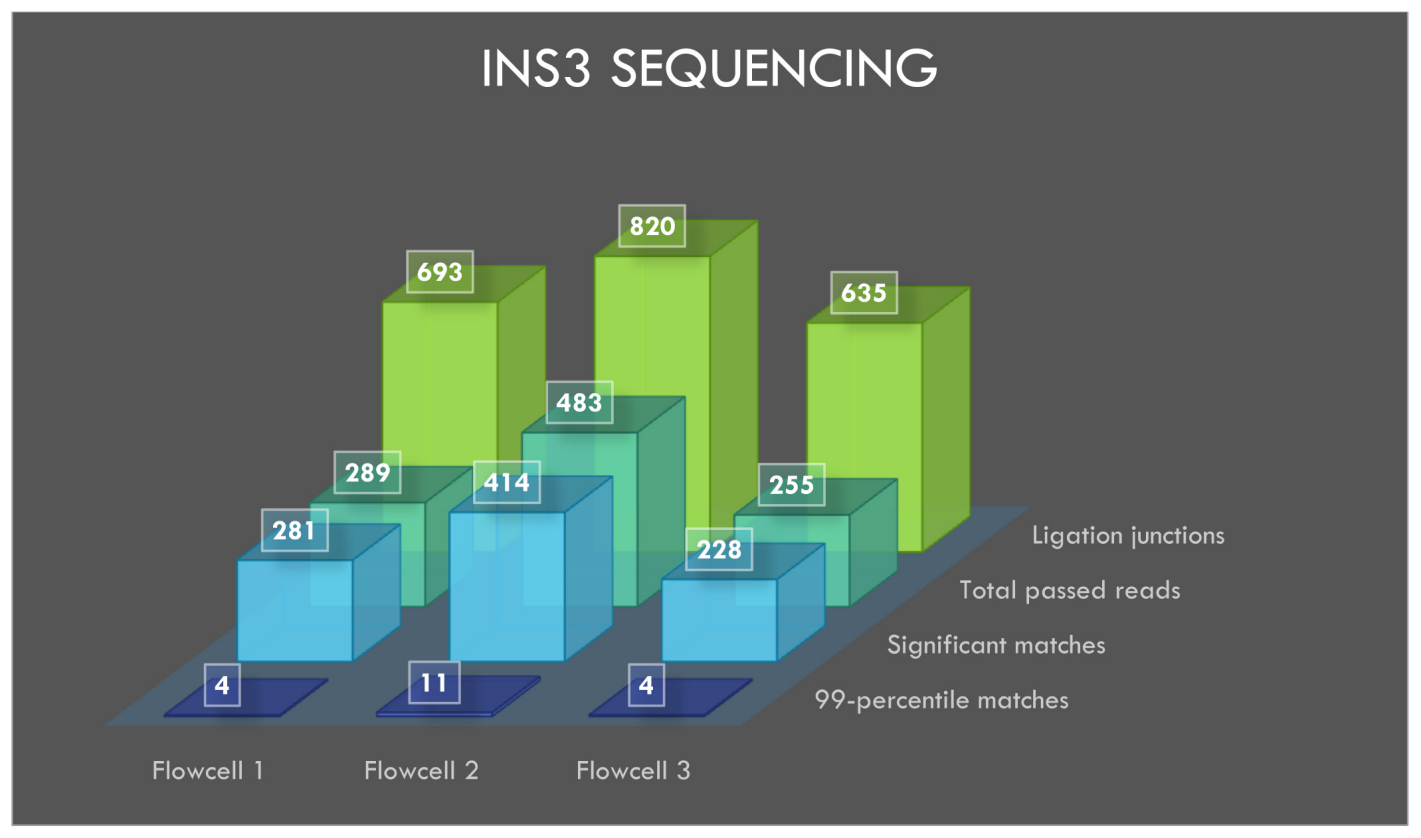
Visualization of INS3 sequencing results.

**Figure 5.**
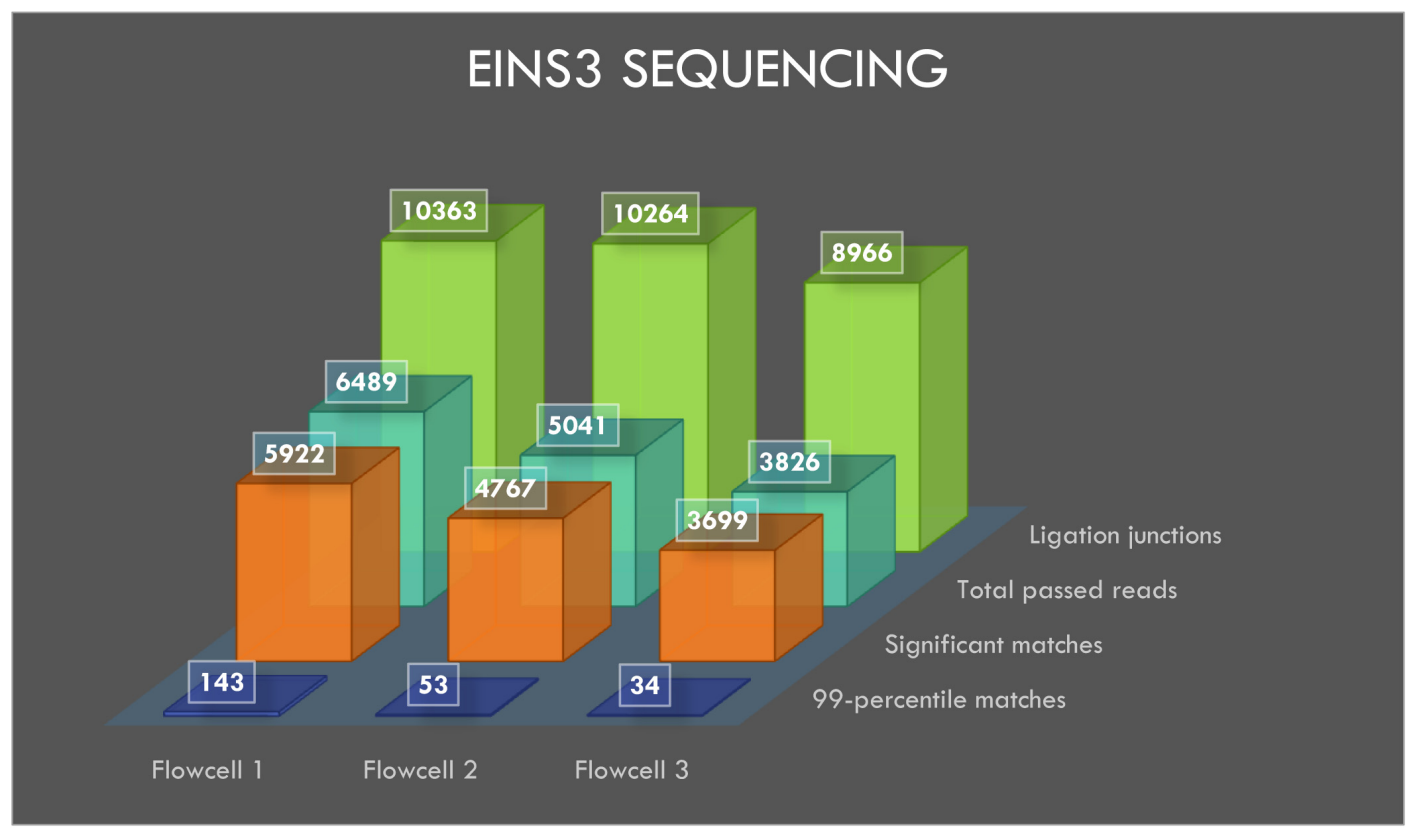
Visualization of EINS3 sequencing results.

**Figure 6.**
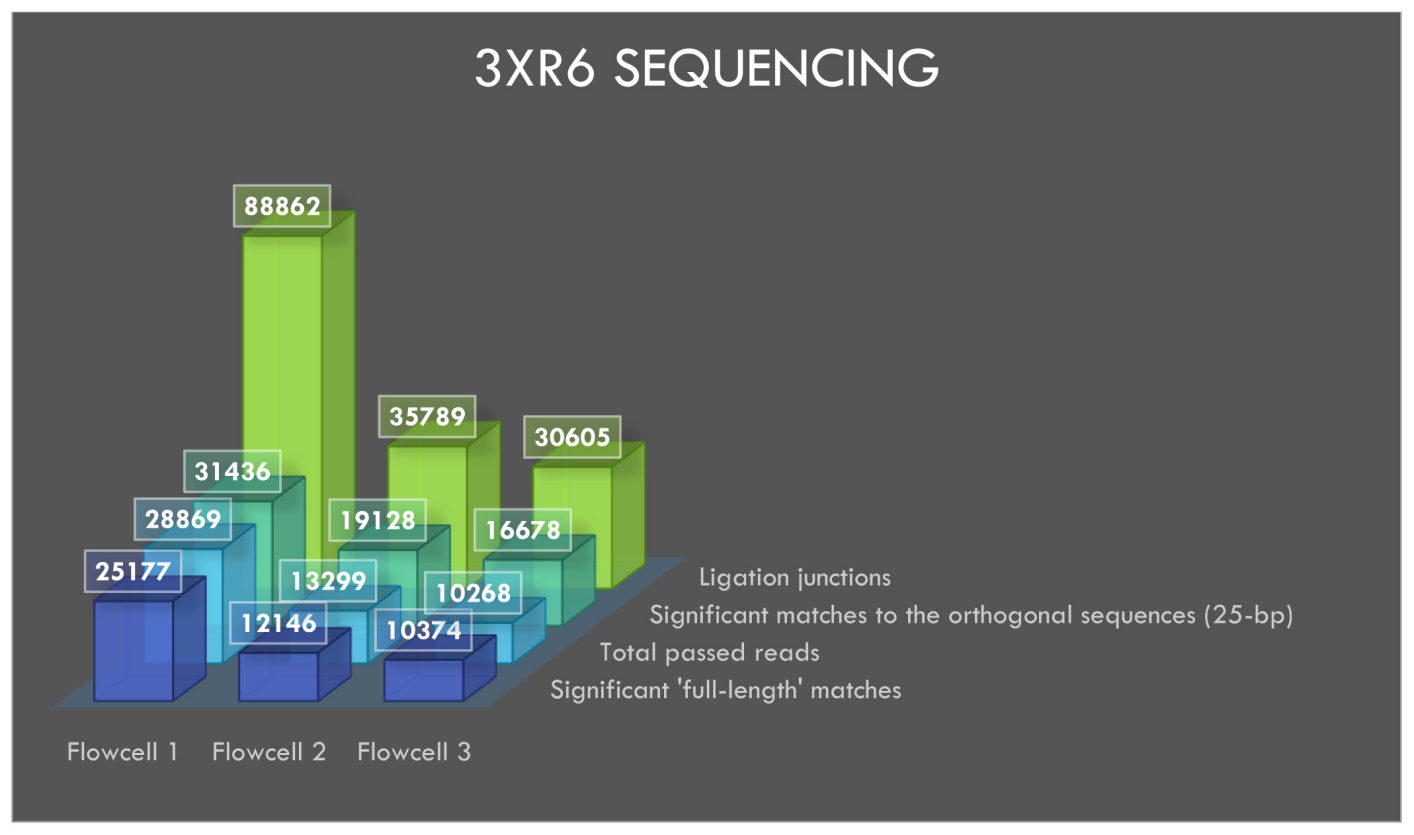
Visualization of 3xr6 sequencing results.

### Ligation junctions and multi-strand reads

**Figure 7.**
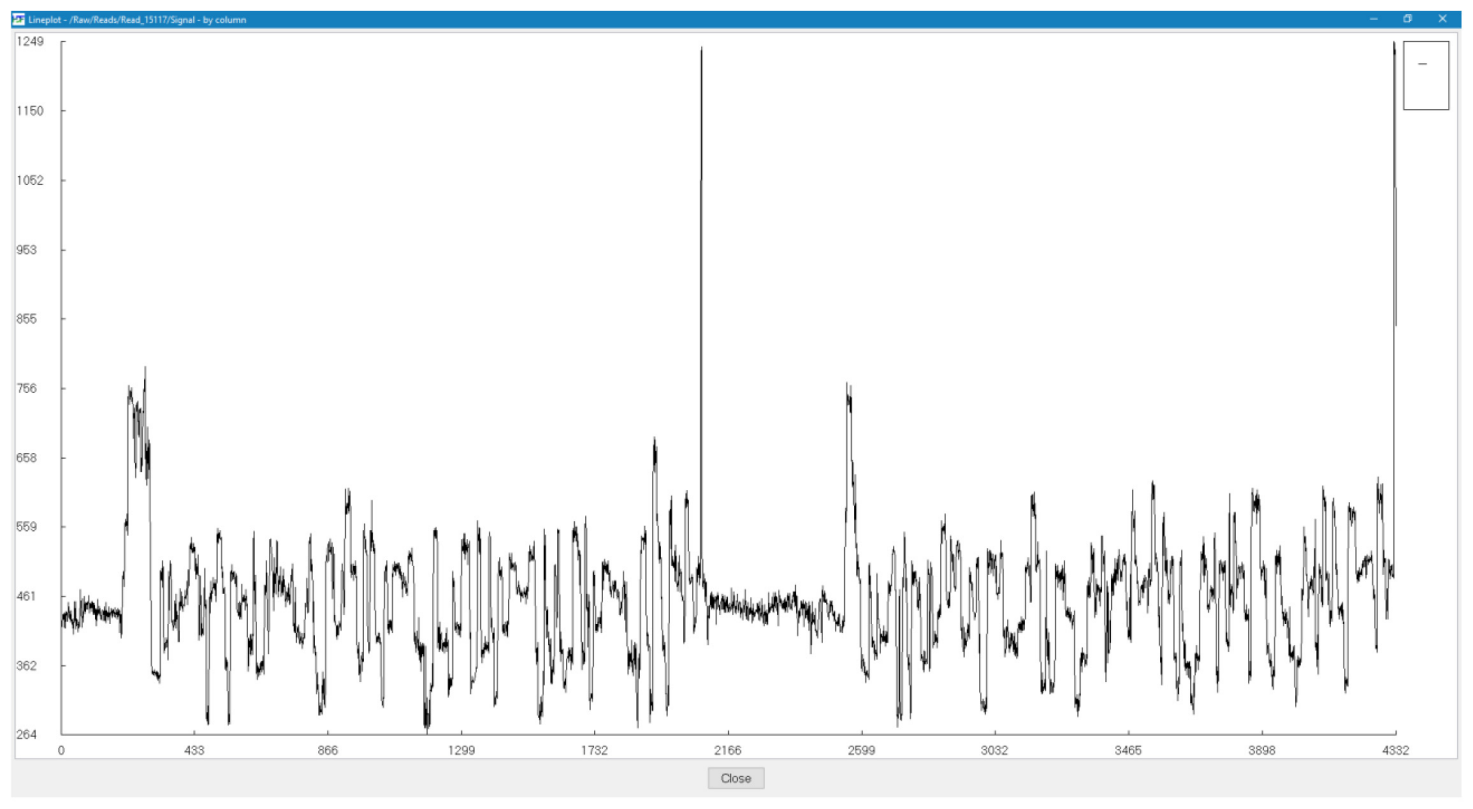
Visualization of a fusion read – [7a6c8a08-3d36-4fcf-a765-ef9cf3e28188] from 3xr6 sequencing (flowcell 1).

The ligation junctions where the AMX adapter binds to the INS3, EINS3 and 3xr6 oligos are searched using BLASTN with ‘blastn-short’ flag. The junction sequences AMXpINS3, AMXpINS3 and AMXpFP as shown in the Table 1, Table 2 and Table 3, respectively, are plotted in Figure 4– Figure 6. The ligation junctions are higher in number than the actual number of reads due to the read artifacts where each read may contain more than one strand. We have identified several of these multi-strand reads, and visualization of such a read is provided in Figure 7.

## Conclusion

We have proposed and implemented a method to sequence single-strand nucleic acid species without performing amplification, second-strand synthesis or a spacer ligation step with helicase-based biological nanopores offered by ONT. Oligonucleotides with a free 3’-OH can also be sequenced successfully with the described method. We have identified sequencing artifacts during our data analysis. Some empty signals can be earmarked as valid reads by the MinION data processing system and reads containing several strands per read are also found, which could be because of segmentation errors.

We believe that this sequencing approach might open new avenues for DNA-based information storage systems and lead to improvements in signal-level data analysis for nanopore sequencing.

## Data availability

### Underlying data

Imperial College London – Research Data Repository: Direct oligonucleotide sequencing with nanopores, https://doi.org/10.14469/hpc/8139 ^10^.

This project contains the following underlying data:

- Raw fast5 files from INS3 sequencing run in 1a.zip, 2b.zip and 3d.zip
- Raw fast5 files from EINS3 sequencing run in eins_1a.zip, eins_2b.zip and eins_3d.zip
- Raw fast5 files from 3xr6 sequencing run in 1a-42k(split).zip, 2b-42k(split).zip and 3d-42k(split).zip
- Basecalled fasta files and BLASTN analysis data from the sequencing runs in data.zip

### Extended data

Imperial College London – Research Data Repository: Direct oligonucleotide sequencing with nanopores, https://doi.org/10.14469/hpc/8139 ^10^.

This project contains the following extended data:

- Supplementary_Table_1.tsv (Supplementary Table 1)

Data are available under the terms of the Creative Commons Attribution 4.0 International license (CC-BY 4.0).
