# Supplementary material for: Direct oligonucleotide sequencing with nanopores
Source: Open Res Eur. 2021 Aug 24;1:47. Originally published 2021 May 12. [Version 2] doi: 10.12688/openreseurope.13578.2 (PMC10445935; doi:10.12688/openreseurope.13578.2)
Supplement: Peer review history

# Peer review: Reviewer response for version 2

**DOI:** 10.21956/openreseurope.15156.r27463

**Published:** 2021-08-31

**Authors:** Fumihito Miura

**Affiliations:** 1 Department of Biochemistry, Kyushu University Graduate School of Medical Sciences, Fukuoka, Japan

The authors have adequately addressed my concerns. I am fully satisfied with the changes and the authors' responses.

Is the rationale for developing the new method (or application) clearly explained?

Yes

Is the description of the method technically sound?

Yes

Are the conclusions about the method and its performance adequately supported by the findings presented in the article?

Partly

If any results are presented, are all the source data underlying the results available to ensure full reproducibility?

Yes

Are sufficient details provided to allow replication of the method development and its use by others?

Yes

Reviewer Expertise:

Genomics, biotechnology, epigenetics

I confirm that I have read this submission and believe that I have an appropriate level of expertise to confirm that it is of an acceptable scientific standard.

# Peer review: Reviewer response for version 2

**DOI:** 10.21956/openreseurope.15156.r27464

**Published:** 2021-08-27

**Authors:** Jeff Nivala, Karen Zhang

**Affiliations:** 1 Paul G. Allen School of Computer Science & Engineering, University of Washington, Seattle, WA, USA; 2 Molecular Information Systems Lab, University of Washington, Seattle, WA, USA

The authors have addressed our concerns.

Is the rationale for developing the new method (or application) clearly explained?

Yes

Is the description of the method technically sound?

Yes

Are the conclusions about the method and its performance adequately supported by the findings presented in the article?

Yes

If any results are presented, are all the source data underlying the results available to ensure full reproducibility?

Yes

Are sufficient details provided to allow replication of the method development and its use by others?

Yes

Reviewer Expertise:

nanopore technologist

We confirm that we have read this submission and believe that we have an appropriate level of expertise to confirm that it is of an acceptable scientific standard.

# Peer review: Reviewer response for version 1

**DOI:** 10.21956/openreseurope.14648.r26969

**Published:** 2021-07-27

**Authors:** Jeff Nivala, Karen Zhang

**Affiliations:** 1 Paul G. Allen School of Computer Science & Engineering, University of Washington, Seattle, WA, USA; 2 Molecular Information Systems Lab, University of Washington, Seattle, WA, USA

This manuscript describes an approach to sequence 5’ phosphorylated ssDNA using commercial nanopore sequencing (ONT). The trick is to anneal an oligo onto the 5’ end of the ssDNA such that there is an A-overhang suitable for TA-based ligation of the dsDNA ONT sequencing adapter onto the ssDNA-oligo complex. Some comments and questions:

- The success of this approach is somewhat foreseeable considering that ONT-based 2D and 1D-squared style sequencing demonstrate the helicase motor can process ssDNA through the pore.
- Overall, the results have been presented clearly for the most part, and interpretation of the results seems sound.
- We agree with the authors, it does simplify the workflow for sequencing of large oligo pools, eliminating the need for PCR.
- It was also interesting that the authors omitted the purification step following adapter ligation, this also simplifies readout workflow considerably.
- Possible related to this is the relatively low read counts, and low number of reads passing filter. It would be great if the authors could discuss this a bit more. Is it possible this is due to committing the cleanup step?
- For those not used to looking at raw nanopore traces, it would help to annotate them with the phenomena that the authors are trying to highlight.

Is the rationale for developing the new method (or application) clearly explained?

Yes

Is the description of the method technically sound?

Yes

Are the conclusions about the method and its performance adequately supported by the findings presented in the article?

Yes

If any results are presented, are all the source data underlying the results available to ensure full reproducibility?

Yes

Are sufficient details provided to allow replication of the method development and its use by others?

Yes

Reviewer Expertise:

nanopore technologist

We confirm that we have read this submission and believe that we have an appropriate level of expertise to confirm that it is of an acceptable scientific standard, however we have significant reservations, as outlined above.

# Peer review: Reviewer response for version 1

**DOI:** 10.21956/openreseurope.14648.r27140

**Published:** 2021-06-30

**Authors:** Fumihito Miura

**Affiliations:** 1 Department of Biochemistry, Kyushu University Graduate School of Medical Sciences, Fukuoka, Japan

The authors presented a method to prepare sequencing libraries for Oxford Nanopore's sequencers from short oligonucleotides by partially forming double-stranded DNA for adapter tagging with dsDNA ligation.

 The ideas and results seem good. However, as a whole, their data presentations and explanations are poor. 

**Major**

1. In Tables 1 and 2, why the numbers of significant matches to INS3 and EINS3 are lower than the AMXpINS3 and AMXpEINS3, respectively? In my understanding, the AMXpINS3 and AMXpEINS3 contain INS3 and EINS3, respectively, and BLASTN uses local alignment. Therefore, BLASTN should detect any partially matched sequences between the reads and INS3 or EINS3. The authors should provide explanations.
2. What did the authors want to explain with Figures 3 and 7? Only raw data is presented in these figures. The authors should give explanations for stalls and helicase dissociations in Figure 3. Also, they should explain what signals indicate the multi-strand reads in Figure 7.
3. In Figures 4, 5, and 6, the authors used bar charts in 3D. However, while they provide the numbers at the top of the bars, the 3D chart is difficult to compare the values. Therefore, they should use 2D charts in these figures.
4. In the Discussion section, the authors mentioned that "We have also identified that the oligonucleotides can even be 3' unblocked if they do not contain a terminal thymine (T)". What did they mean by this sentence? I could not find any relevant description and data in the Result section for this description.
5. In the last part of the Discussion section, the authors mentioned that "reads containing several strands per read are also found, which could be because of segmentation errors." What did they mean with the "segmentation errors." The authors meant the base caller crash? Or simply program error?

**Minor**

1. What is the "AMX sequencing adaptor" in the Introduction section? While I can find the explanation in the Discussion section, the authors should explain it the place first appeared.
2. There is no explanation for AMXpINS3 and AMXpEINS3.

Is the rationale for developing the new method (or application) clearly explained?

Yes

Is the description of the method technically sound?

Yes

Are the conclusions about the method and its performance adequately supported by the findings presented in the article?

Partly

If any results are presented, are all the source data underlying the results available to ensure full reproducibility?

Yes

Are sufficient details provided to allow replication of the method development and its use by others?

Yes

Reviewer Expertise:

Genomics, biotechnology, epigenetics

I confirm that I have read this submission and believe that I have an appropriate level of expertise to confirm that it is of an acceptable scientific standard, however I have significant reservations, as outlined above.

# Response

**Published:** 2021-07-19

**Authors:** Sachin Chalapati

**Affiliations:** Helixworks Technologies, Ireland

We agree to the points that the reviewer has made and will make the appropriate revisions to the relevant sections, nevertheless we would like to clarify on them in this comment for reference.  

1. AMXpINS3 and AMXpEINS3 are short sequences containing the 3’ end of the sequencing adaptor (AMX) and the 5’ end of INS3 and EINS3 respectively. They are short sequences that do not contain the entirety of INS3 and EINS3 and were found in excess compared to the significant matches of INS3 and EINS3.
2. We have found the helicase dissociation events and multi-strand reads in raw fast5 files, but the data is inconsistent on why they are occurring and the root-cause of these issues. It was observed that in some instances, there can be reads without any valid sequencing data, or instances where there are several sequences within a single fast5 read. We believe that we can rescue the multi-strand reads by splitting them into individual strands and basecalling them. Relevant information would be added to the discussion section.
3. We agree with the reviewer’s suggestion and make changes to the charts.
4. The INS3 and EINS3 strands have a blocked 3’ ends, as to eliminate any spurious ligations taking place during the adaptor ligation step of the library prep. The 3xr6 oligo pool do not have any 3’ end modifications but were sequenced successfully with the same protocol. We were suggesting that the 3’ end can be unblocked and still be sequenced. The line will be corrected appropriately.
5. We believe the multi-strand reads occur due to segmentation issues on the instrument (MinION), where the sequencer is failing to split a read correctly. In general, the nanopore reads would only contain one sequence per read.
6. Information on the sequencing adaptor and other short sequences would be added to the relevant sections.
